# Trajectory of Antidepressant Effects after Single- or Two-Dose Administration of Psilocybin: A Systematic Review and Multivariate Meta-Analysis

**DOI:** 10.3390/jcm11040938

**Published:** 2022-02-11

**Authors:** Chia-Ling Yu, Chih-Sung Liang, Fu-Chi Yang, Yu-Kang Tu, Chih-Wei Hsu, Andre F. Carvalho, Brendon Stubbs, Trevor Thompson, Chia-Kuang Tsai, Ta-Chuan Yeh, Szu-Nian Yang, Jae Il Shin, Che-Sheng Chu, Ping-Tao Tseng, Kuan-Pin Su

**Affiliations:** 1 Department of Pharmacy, Chang-Gung Memorial Hospital, Linkou, Taoyuan 333, Taiwan; beautyarielyou@gmail.com; 2 Department of Psychiatry, Beitou Branch, Tri-Service General Hospital, National Defense Medical Center, Taipei 112, Taiwan; lcsyfw@gmail.com (C.-S.L.); ysn56725@ms4.hinet.net (S.-N.Y.); 3 Graduate Institute of Medical Sciences, National Defense Medical Center, Taipei 114, Taiwan; 4 Department of Neurology, Tri-Service General Hospital, School of Medicine, National Defense Medical Center, Taipei 114, Taiwan; fuji-yang@yahoo.com.tw (F.-C.Y.); jiakuang@office365.ndmctsgh.edu.tw (C.-K.T.); 5 Institute of Epidemiology & Preventive Medicine, College of Public Health, National Taiwan University, Taipei 100, Taiwan; yukangtu@ntu.edu.tw; 6 Department of Psychiatry, Kaohsiung Chang Gung Memorial Hospital, Chang Gung University College of Medicine, Kaohsiung 833, Taiwan; harwicacademia@gmail.com; 7 IMPACT (Innovation in Mental and Physical Health and Clinical Treatment) Strategic Research Centre, School of Medicine, Barwon Health, Deakin University, Geelong, VIC 3220, Australia; andrefc7@hotmail.com; 8 Department of Psychological Medicine, Institute of Psychiatry, Psychology and Neuroscience, King’s College London, London SE5 8AF, UK; brendon.stubbs@kcl.ac.uk (B.S.); cobolsu@gmail.com (K.-P.S.); 9 Physiotherapy Department, South London and Maudsley NHS Foundation Trust, London SE13 6QJ, UK; 10 Centre of Chronic Illness and Ageing, University of Greenwich, London SE10 9LS, UK; t.thompson@greenwich.ac.uk; 11 Department of Psychiatry, Tri-Service General Hospital, National Defense Medical Center, Taipei 114, Taiwan; nsds7520@gmail.com; 12 Department of Psychiatry, Armed Forces Taoyuan General Hospital, Taoyuan 323, Taiwan; 13 Graduate Institute of Health and Welfare Policy, National Yang Ming University, Taipei 112, Taiwan; 14 Department of Pediatrics, Yonsei University College of Medicine, Seoul 03722, Korea; shinji@yuhs.ac; 15 Department of Psychiatry, Kaohsiung Veterans General Hospital, Kaohsiung 813, Taiwan; 16 Center for Geriatric and Gerontology, Kaohsiung Veterans General Hospital, Kaohsiung 813, Taiwan; 17 Non-Invasive Neuromodulation Consortium for Mental Disorders, Society of Psychophysiology, Taipei 114, Taiwan; 18 Graduate Institute of Medicine, College of Medicine, Kaohsiung Medical University, Kaohsiung 807, Taiwan; 19 Prospect Clinic for Otorhinolaryngology & Neurology, Kaohsiung 811, Taiwan; 20 Department of Psychology, College of Medical and Health Science, Asia University, Taichung 413, Taiwan; 21 Institute of Biomedical Sciences, National Sun Yat-sen University, Kaohsiung 804, Taiwan; 22 Mind-Body Interface Laboratory (MBI-Lab), China Medical University and Hospital, Taichung 404, Taiwan; 23 An-Nan Hospital, China Medical University, Tainan 709, Taiwan

**Keywords:** depression, meta-analysis, psilocybin, psychedelics

## Abstract

We examined the cardiovascular safety, acceptability, and trajectory of the antidepressant effects of psilocybin after single- or two-dose administration. Four major electronic databases were systematically searched. Data were pooled using a multivariate random-effects meta-analysis. Primary outcomes were changes in depressive symptoms. Secondary outcomes were cardiovascular safety and acceptability. Ten studies were included. The estimated effect sizes (standardized mean difference (SMD) with 95% confidence intervals) for psilocybin were −0.75 (−1.15 to −0.35) on day 1, −1.74 (−2.15 to −1.32) at 1 week, −1.35 (−1.77 to −0.93) at 1 month, −0.91 (−1.31 to −0.51) at 3 months, and −1.12 (−1.56 to −0.68) at 6 months. Higher doses and two sessions of psilocybin treatment were significantly associated with superior antidepressant effects. The all-cause discontinuation and heart rate after psilocybin administration were comparable to placebo; meanwhile, psilocybin increased systolic and diastolic blood pressure by 19.00 mmHg and 8.66 mmHg, respectively. There were no significant differences between SMD derived from placebo-controlled trials compared to those from pre–post changes and SMD in randomized controlled trials (RCTs) compared to those in non-RCTs. The present study demonstrates that single- or two-dose psilocybin administration has rapid and sustained antidepressant effects for up to 6 months, with favorable cardiovascular safety and acceptability.

## 1. Introduction

Psilocybin is a serotonergic hallucinogen that undergoes rapid dephosphorylation into psilocin. It is a 5-HT2a receptor agonist that causes distortions in perception, thoughts, and emotions, as well as self-boundary dissolution [1]. In the 1950s and the 1960s, psilocybin was used to treat psychological distress in clinical trials; in the 1970s, with the enactment of the Controlled Substances Act, its use was discontinued, and it was classified as a Schedule I drug [2]. With the increasing understanding of the molecular and neurobiological mechanisms of psychedelics, clinical and research interest in such agents as novel therapeutic targets for the management of mental disorders has steadily grown since the 1990s [3]. In 2008, a guideline was developed to ensure the safety of studies using psychedelics as a treatment for mental disorders [4]. One of the most commonly reported side effects of psychedelics was an acute sympathetic response in dynamic blood pressure and heart rate. Another potential long-term risk is hallucinogen persisting perception disorder (HPPD), which is defined as chronic perceptual changes for a few minutes to several months that can interfere with daily functioning and reduce quality of life and satisfaction [5].

Psilocybin may exert psychoactive effects through 5-HT2a receptor agonism. Serotonin 5-HT2a receptors are widely distributed in the frontal cortex and hippocampus, which are key regions involved in brain networks involved in learning, cognition, and emotional regulation [6]. In animal studies, 5HT2a receptor agonists enhanced the cognitive flexibility and response inhibition of individuals [7]. In addition, this mechanism has been associated with antidepressant-like effects in animal models [8]. Intravenous administration of psilocybin significantly decreases the positive coupling of the medial prefrontal cortex and the posterior cingulate cortex, and these brain regions are known to be involved in patients with depression. This neurobiological effect may be a neuroimaging biosignature related to the effectiveness of antidepressant treatments [9].

Classic serotonergic psychedelics include psilocybin, lysergic acid diethylamide (LSD), and ayahuasca. Several meta-analyses have explored the putative role of psychedelics in the management of mental disorders. However, these studies included both healthy participants and patients with a variety of mental disorders, such as depression and/or anxiety, post-traumatic stress disorder, cancer-related mood disorders, and even pain [10,11,12,13,14,15,16]. These meta-analyses suggested that psychedelics exert antidepressant effects, that they are pleiotropic compounds, and that combining all serotonergic psychedelics may not be methodologically appropriate [17]. Two meta-analyses specifically investigated the antidepressant/anxiolytic effects of psilocybin [11,16]. However, several new clinical trials [18,19,20,21,22] have recently been published and are not included in these meta-analyses [10,11,16]. Previously published meta-analyses only considered pre–post changes in depressive symptoms, therefore potentially missing important information regarding the trajectory of improvements in terms of these symptoms. 

The current study aimed to examine the trajectory of the antidepressant effects of psilocybin, which may help determine the promptness and duration of the antidepressant effects of psilocybin after single- or two-dose administration. We also sought to evaluate the cardiovascular safety and acceptability of psilocybin. As measurements were performed at multiple time points, we conducted a multivariate meta-analysis, taking into account correlations between effect sizes at different time points. We also explored potential sources of heterogeneity across studies.

## 2. Materials and Methods

We performed a systematic review and meta-analysis of clinical trials examining the efficacy of psilocybin in reducing depressive symptoms. This study complied with the Preferred Reporting Items for Systematic Reviews and Meta-analyses (PRISMA) 2020 statement [23] (Supplementary File S1). This study followed an a priori developed protocol that is registered with the International Prospective Register of Systematic Reviews (PROSPERO) and is available online (www.crd.york.ac.uk/prospero, CRD42021252492, accessed date on 27 May 2021).

### 2.1. Data Sources and Searches

We systematically searched the MEDLINE, Cochrane Central Register of Controlled Trials, Embase, and PsycINFO databases from the time of their inception until 27 January 2021 to identify all clinical trials reporting the efficacy of psilocybin in the management of depressive symptoms. Further studies were identified by manually searching the reference lists of eligible studies, as well as those included in previous meta-analyses [10,11,12,13,14,15,16]. Supplementary File S1 presents the details of the search strategy. 

### 2.2. Study Selection

Two investigators (C.-L. Yu and C.-S. Liang) independently screened the titles, abstracts, and full texts of potentially eligible references. All clinical trials concerning psilocybin were considered eligible for inclusion if the primary outcome was the assessment of the antidepressant effects of psilocybin. Because depressive symptoms can also occur in patients with physical disorders, we included not only patients with major depressive disorders (MDDs) but also those with physical disorders comorbid with depression. We excluded case reports and case series (*n* < 10). Gray literature (e.g., conference abstracts) and protocols were also excluded. Discrepancies regarding study inclusion were resolved through discussion with additional input from a third investigator (C.-S. Chu).

### 2.3. Outcome Measures

The outcomes of interest were specified a priori based on recent meta-analyses [11,16]. In the current study, we focused on changes in depressive symptoms at different follow-up time points after either single- or two-dose administration of psilocybin, with the aim of exploring the trajectory of antidepressant effects. The primary outcomes were changes in depressive symptoms on day 1, week 1, month 1, month 3, and month 6. The changes in depressive symptoms were as follows: (1) differences between psilocybin and placebo in pre–post changes in depressive symptoms or (2) pre–post changes in depressive symptoms in the absence of a placebo arm. The secondary outcomes were all-cause discontinuation (acceptability) and cardiovascular safety profiles (including peak systolic blood pressure (SBP), diastolic blood pressure (DBP), and heart rate (HR)) compared with placebo treatment on the day of administration.

### 2.4. Data Extraction and Risk of Bias Assessment

Two authors (Ta-Chuan Yeh and Ping-Tao Tseng) independently abstracted aggregate-level data for each included study using a prespecified data extraction form and appraised the risk of bias of each included trial using the Cochrane Risk of Bias Tool [24]. The extracted data included (i) study design, total number of participants, sociodemographic data of participants enrolled in each trial, and follow-up period; (ii) intervention details (e.g., single- or two-dose psilocybin administration), and (iii) outcome measures. If information regarding average body weight was unavailable, a body weight of 70 kg was used to calculate the dose of psilocybin (mg/kg). For SBP, DBP, and HR, we extracted the peak changes involving these outcomes. If relevant data were only provided in figures, WebPlotDigitizer was used for data extraction. Discrepancies in data abstraction and risk of bias assessment were resolved either through arrival at a consensus or with input from a third investigator (Chu C.-S.). We also contacted the relevant corresponding authors to request the necessary data if these were unavailable in their original article.

### 2.5. Data Synthesis

All statistical analyses were conducted using R Project (v.4.0.3, R Foundation) and STATA version 16.0 (StataCorp LLC Statistics/Data Analysis StataCorp, College Station, TX, USA). Pooled effect sizes for primary and secondary outcomes were estimated using a random-effects meta-analysis with restricted maximum likelihood estimation. We computed the standardized mean differences (SMDs) for primary outcomes; mean differences for HR, SBP, and DBP; and odds ratios (ORs) for acceptability. Negative changes in depressive symptoms indicate improvement in depressive symptoms. If different published papers from the same trial reported different effect sizes and different sample sizes, we subtracted small ones from large ones, eliminating the chance of redundant subjects. For studies involving two-dose psilocybin administration, we also extracted the effect sizes of single-dose administration. We fitted the five time points of measurements into a multivariate model in R using the *metaphor* package, taking into account heterogeneity and dependency involving the true underlying effects at multiple time points. Standard pairwise meta-analyses of secondary outcomes were performed. Heterogeneity was summarized using estimates of between-study variation (τ^2^), and the proportion of variability in effect estimates due to between-study heterogeneity was summarized using the I^2^ statistic. Substantial heterogeneity involving changes in depressive symptoms was expected because we fitted five time points of measurements into a single multivariate model.

### 2.6. Meta-Regression and Subgroup Analysis

We conducted several preplanned meta-regression and subgroup analyses to examine potential moderators of primary outcomes, including psilocybin dose, number of participants, age, proportion of women, study duration, participants with MDDs, and patients with cancer. Studies encompassed reports of two doses of psilocybin versus those using a single dose of psilocybin, participants with severe depressive symptoms vs. those without severe depressive symptoms (defined based on the rating scale used), psilocybin treatment combined with psychotherapy vs. psilocybin treatment without concurrent psychotherapy, randomized controlled trials (RCTs) vs. non-randomized controlled trials (NRCTs), and SMD derived from placebo-controlled trials vs. SDM derived from pre–post changes. Meta-regression and subgroup analyses were performed using a multivariate meta-analytic model if the included studies were >10. Bubble plots were generated for statistically significant moderators.

### 2.7. Publication Bias and Sensitivity Analysis

For publication bias, 1-way sensitivity analysis, and influence analyses, we aggregated all time points of effect sizes into a single effect size for each study. Publication bias was assessed using funnel plots and Egger’s regression tests for primary outcomes. We conducted 1-way sensitivity analyses to determine the robustness of the findings for the primary outcomes. A series of influence analyses were performed to detect potential outlier studies based on different influence measures, including standardized residuals, Cook’s distance, τ^2^, and hat value [25]. A Baujat plot was drawn to determine studies that overly contributed to the heterogeneity of primary outcomes [26]. We also conducted a series of multivariate meta-analyses to examine the trajectory of the antidepressant effects of psilocybin by using different subsets of data (Table S1). 

Robust variance estimation and nonlinear models were also employed for further sensitivity analyses. Robust variance estimation methods provide a way to include all dependent effect sizes in a single meta-regression model, even when the nature of that dependence is unknown and the sample size is small [27]. In the primary multivariate model, time was included as a factor, and the coefficients of the five time points were examined by robust variance estimation in R using the *clubsandwich* package. We fitted three additional multivariate models to determine the best-fitted models: (i) linear model using time (month) as a continuous variable, (ii) nonlinear model using time and time^2^, and (iii) nonlinear model using restricted cubic spline.

## 3. Results

**Figure 1:**
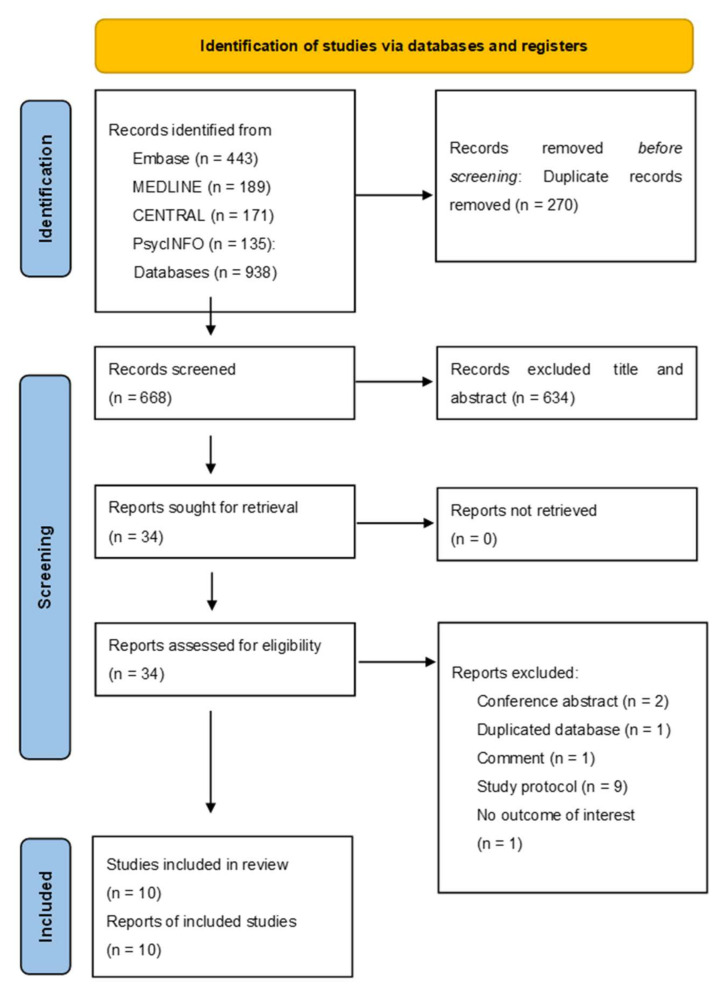
PRISMA 2020 flow diagram for new systematic reviews that included searches of databases and registers only.

Overall, 938 unique references were identified after searching multiple databases (Supplementary File S2). The PRISMA flow chart (Figure 1) shows that 668 articles were excluded after screening the title, abstract, and duplicated records; 344 articles were scrutinized, and 14 were excluded for certain reasons (Supplementary File S3). Finally, 10 studies (Table 1) published between 2011 and 2020 met the inclusion criteria [18,19,20,21,22,28,29,30,31,32]. Of these, five were open-label clinical trials [19,21,22,28,29], four were randomized controlled studies [20,30,31,32], and one was a post-RCT follow-up study [18]. Table 1 provides the demographic and clinical characteristics of the included studies. A total of 208 participants were included, with a mean age of 48.4 years (standard deviation = 7.0) and a mean proportion of women of 44.3%. With regard to the study population, five trials included patients with MDDs [20,21,22,28,29], four included patients with cancer [18,30,31,32], and one included patients with HIV/AIDS [19]. Five trials used a single dose of psilocybin [18,19,30,31,32], while five used two doses [20,21,22,28,29].

### 3.1. Quality Assessment

Among the RCTs, two [31,32] were judged to have a high risk of bias (ROB) because of the domain of blinding of outcome assessment (Figure S2). The high ROB in each domain ranged from 0 to 50.0% among the RCTs. Among the 10 studies, 8 had a high ROB, with blinding of outcome assessment being the most frequent (Figure S3). The risk of bias in each domain ranged from 0 to 70.0%.

### 3.2. Primary Outcome: Depressive Symptoms

**Figure 2:**
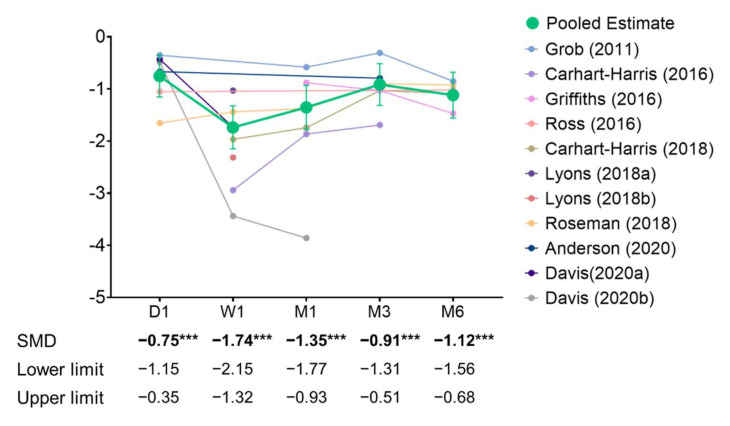
Trajectory of antidepressant effects of psilocybin. *** *p* < 0.001.

From day 1 to month 6, single- or two-dose psilocybin treatment was significantly associated with reduced depressive symptoms (Figure 2). The estimated effect sizes were moderate to large on day 1 (5 studies, SMD = −0.75, 95% confidence interval (CI): −1.15, −0.35) and large at week 1 (5 studies, SMD = −1.74, 95% CI: −2.15, −1.32), month 1 (6 studies, SMD = −1.35, 95% CI: −1.77 to −0.93), month 3 (6 studies, SMD = −0.91, 95% CI: −1.31 to −0.51), and up to month 6 (5 studies, SMD = −1.12, 95% CI: −1.56 to −0.68).

### 3.3. Meta-Regression and Subgroup Analyses of Primary Outcomes

**Figure 3:**
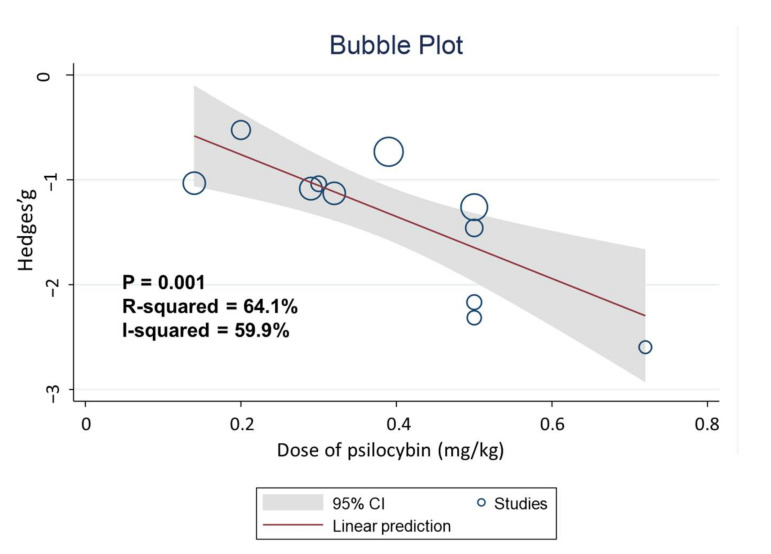
Bubble plot for dose of psilocybin and Hedges’ g.

Higher doses of psilocybin were associated with a greater reduction in depressive symptoms (slope = −1.89, *p* = 0.02) than administration of a lower psilocybin dose and contributed to 64.1% of the changes in depression severity, with moderate heterogeneity (R^2^ = 64.1%, I^2^ = 59.9%) (Figure 3). In addition, two-dose psilocybin administration also contributed to a greater reduction in the severity of depression (Table 2) compared with single-dose psilocybin administration (slope = −0.50, *p* = 0.049). Among studies that reported two-dose psilocybin administration, four had a treatment interval of 7 days [21,22,28,29], while one had an average treatment interval of 1.6 weeks [20]. Other moderators and the results of subgroup analyses were not significant (Table 2), including RCT vs. non-RCT (*p* = 0.46) and SMD derived from placebo-controlled trials vs. SMD derived from pre–post changes (*p* = 0.37).

### 3.4. Publication Bias and Sensitivity Analyses

**Figure 4:**
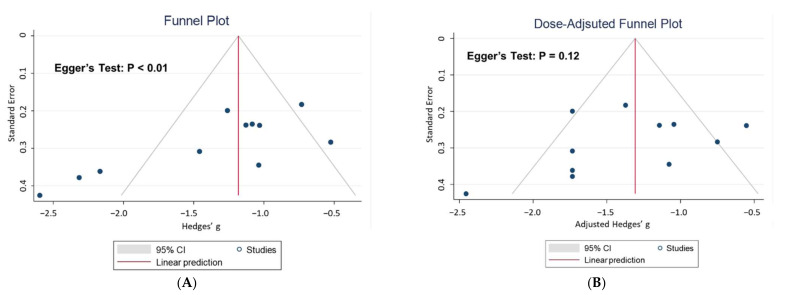
(**A**) Funnel plot with Egger’s test. (**B**) The dose-adjusted funnel plot.

Figure 4A illustrates the small study effects for primary outcomes determined using Egger’s test (*p* < 0.01). However, the dose-adjusted funnel plot (Figure 4B) did not show any publication bias (*p* = 0.12). The effects of psilocybin on depression severity remained robust in the one-study removal tests (Figure S4). The Baujat plot (Figure S5) examined which studies contributed most to the heterogeneity and overall influence on the results compared to others, showing that the study by Anderson et al. [19] contributed to a greater influence on the results and had a greater contribution to heterogeneity. The diagnostic influence analyses for outliers of the included studies showed that none of them was considered an outlier, which can be measured by standardized residuals, Cook’s distance, tau-squared, hat values, DFFTIS value (indicating (in standard deviations) how much the predicted pooled effect changes after excluding a particular study), covariance ratio, and Q statistic (Figures S6–S13).

**Figure 5:**
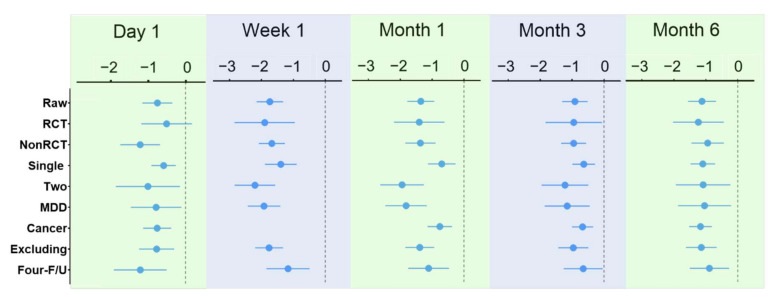
Results of sensitivity analyses.

A subset of data was extracted for other multivariate meta-analyses (Figure 5 and Table S1), including RCTs, NRCTs, single-dose studies, repeated-dose studies, MDD studies, and cancer studies, excluding outlier studies [19], and studies with ≥4 time-point measurements. The results of these studies showed that the antidepressant effects of psilocybin remained significant after single- or two-dose administration on day 1, week 1, month 1, month 3, and month 6. When looking at the results of RCTs (Table S1), psilocybin treatment was not significantly associated with reduced depressive symptoms on day 1 (SMD = −0.50, 95% CI: −1.18 to 0.17) but was significantly associated with reduced depressive symptoms by week 1 (SMD = −1.90, 95% CI: −2.84 to −0.84), month 1 (SMD = −1.40, 95% CI: −2.20 to −0.20), month 3 (SMD = −0.95, 95% CI: −1.83 to −0.806), and month 6 (SMD = −1.23, 95% CI: −2.03 to −0.03). 

Finally, robust variance estimation confirmed the findings of our original multivariate meta-analytic model (Table 2). In addition, this model fitted better than the linear (time as continuous variable), quadratic (time plus time^2^), and restricted cubic spline models (Table S3). 

### 3.5. Secondary Outcomes: All-Cause Discontinuation, SBP, DBP, and HR Compared with Placebo

Psilocybin treatment was significantly associated with elevated SBP and DBP compared to placebo treatment. Compared with placebo, psilocybin treatment was associated with an increase in SBP of 13.58 mmHg to 24.41 mmHg, with an average increase of 19.00 mmHg. Compared with placebo, psilocybin treatment was associated with an increase in DBP of 5.18 mmHg to 12.15 mmHg, with an average increase of 8.66 mmHg. Compared with placebo, no significant difference was found in all-cause discontinuation and HR following psilocybin administration (Figure S1).

## 4. Discussion

This meta-analysis investigated the trajectory of the antidepressant effects of psilocybin from day 1 to month 6 after single- or two-dose administration. The main findings of this study were as follows. First, we observed a significant moderate-to-large effect size of antidepressant effects on day 1. Second, the antidepressant effects of psilocybin were sustained, with a substantial effect size from 1 week to 6 months after administration. Third, patients receiving a higher dose of psilocybin or two-dose administration showed superior improvements in terms of depressive symptoms compared with those who received a lower or single-dose administration. Fourth, psilocybin demonstrated cardiovascular safety, was well tolerated, and resulted in increases in SBP and DBP levels of 19.00 mmHg and 8.66 mmHg, respectively. However, the cardiovascular effects of psilocybin are self-limiting.

Patients exhibited a rapid reduction in depressive symptoms one day after the administration of psilocybin. This finding is clearly different from the onset of antidepressant effects of traditional antidepressants, which usually occur after administering a daily dose of antidepressant drugs for at least two weeks [33]. In a head-to-head RCT trial of psilocybin vs. escitalopram in patients with moderate to severe MDD, no significant difference was found in the antidepressant effects between psilocybin and escitalopram [34]. However, the sustained antidepressant effects (at week 6) after a single dose of psilocybin may alleviate the daily dosing burden and improve treatment adherence in patients with affective disorders (median prevalence of 40%) treated with traditional antidepressants [35]. Moreover, this RCT [34] reported a more rapid onset of the antidepressant effects of psilocybin vs. escitalopram at weeks 1 and 2. The rapid therapeutic effects of psilocybin, one of the “psychedelics” (a Greek word for “mind revealing”), are not solely due to its pharmacological action but rather are subjective and experience-dependent [22]. Psilocybin sessions can produce profound psychological “peaks” or “mystical experiences,” characterized by a sense of meaningfulness, insightfulness, and unity [36]. The quality and intensity of the acute psychological experience can be predictors of medium- and long-term psychological health and clinical outcomes [22]. 

The present meta-analysis identified that psilocybin had a large effect size in reducing depressive symptoms by week 1, which is consistent with ketamine (with the strongest response occurring between weeks 1 and 2 after administering the medication) [37]. Furthermore, recent meta-analyses have revealed large effect sizes of psychedelics in reducing depressive symptoms on days 2 and 15 [10] and on days 7 and 21 [14]. The trajectory of antidepressant effects was observed to commence one day after psilocybin administration, and the therapeutic effects were sustained for up to six months, even with only a single or two doses of psilocybin.

Another interesting finding was that patients who received higher doses of psilocybin or the two-dose regimen exhibited better improvement in terms of depressive symptoms than those who received lower doses of psilocybin or the single-dose regimen. Higher doses can contribute to greater subjective drug effects, such as mystical experiences and altered consciousness [14], which might play an important role in improving the efficacy of psilocybin. Currently, there remains a lack of data regarding the optimal dose and the appropriate number of doses of psilocybin, the identification of which is necessary to improve its efficacy and safety. Greater hemodynamic changes were observed in patients with a body mass index of 30 or higher receiving ketamine. This result implies that body mass index may affect the optimal dose [38]; however, neither weight-adjusted nor fixed-dosing of psilocybin showed a significant impact on subjective drug effects with clinical relevance [39]. Moreover, whether more than two doses of psilocybin administration could increase its efficacy as a treatment for depression remains unknown. In a study recruiting healthy participants, no differences were observed in mystical experiences between patients who received three doses of psilocybin and those who received a single dose [40]. In another LSD study using healthy participants, the dose–response curve for LSD showed a ceiling effect for positive subjective effects, whereas ego distortion with anxiety was observed in patients receiving a higher dose [41]. Therefore, further studies are needed to identify appropriate dosing schedules so as to achieve maximum beneficial effects without producing extreme perceptual distortions.

Our study had a number of limitations. First, we included NRCTs; therefore, large effect sizes may be related to pre–post changes in depressive symptoms. In addition, the open-label nature of these studies may have confounded the results. However, the results of RCTs showed that psilocybin treatment was significantly associated with reduced depressive symptoms from week 1 to month 6, and there was no significant difference between SMD derived from placebo-controlled trials and SMD derived from pre–post changes. Second, two of the four RCTs had a high ROB in the blinding domain. “Active” placebos may be an appropriate method. For example, a recent RCT used a very low dose of psilocybin (1 mg) as an “active” placebo [34]. A methodological design can be used in future studies. Third, few studies have established a diagnosis of depression using a validated structured/semi-structured interview. Fourth, the sample sizes of the included studies were relatively small (range *n* = 12–56), and the trajectory of the antidepressant effects of psilocybin needs to be explored further. More studies involving larger sample sizes or longer follow-up periods are needed to validate our findings. Fifth, we did not examine the long-term health impacts of psilocybin, such as HPPD and psychosis [5], due to unavailable data. Future studies examining these long-term side effects are warranted. 

## 5. Conclusions

The current meta-analysis demonstrated that psilocybin treatment could contribute to a rapid and sustained improvement in depressive symptoms. Higher doses and a two-dose regimen of psilocybin exhibited greater efficacy; moreover, psilocybin was relatively safe and well tolerated. Although we provide the most comprehensive evidence of the antidepressant trajectory of psilocybin, additional, well-designed RCTs are warranted to confirm or refute the tentative findings provided herein.

## Figures and Tables

**Table 1:** Demographic and clinical characteristics of the included studies.

Study	Dx	Sample Size	Age (Years)	Female	Depression Severity	Dosing	Psychiatric Comorbidities	Study Design	Dropout	Serious AE
**Grob 2011**	Cancer	12	36–58	91.6%	Mild; BDI ^a^, active arm, 16.1; placebo arm, 14.5	Oral single dose, 0.20 mg/kg	Yes. Specific psychiatric disorders were not mentioned	Double-blind RCT	0	No
**Carhart-Haris 2016**	MDD (TRD)	12	42.7 (10.2)	50%	Severe; BDI, 33.7	Oral two doses, 10 mg and 25 mg, 7 days apart	Excluding psychotic disorder, serious suicide attempts, mania, and drug or alcohol dependence	Open-label single-arm trial	0	No
**Griffiths 2016**	Cancer	56	56.3 (10.0)	49.0%	Mild; BDI, active arm, 17.7; placebo arm, 18.4	Oral single dose, 22 or 30 mg	All participants had a psychiatric disorder, including adjustment disorder, dysthymia, GAD, or MDD	Double-blind RCT	5	No
**Ross 2016**	Cancer	31	56.3 (12.9)	62.1%	Mild; BDI, active arm, 15.0; placebo arm, 16.8	Oral single dose, 0.3 mg/kg	Adjustment disorder and GAD	Double-blind RCT	3	No
**Carhart-Haris 2018**	MDD (TRD)	20	44.0 (11.0)	30%	Severe; BDI, 34.5	Oral two doses, 10 mg and 25 mg, 7 days apart	Excluding psychotic disorder, serious suicide attempts, mania, and drug or alcohol dependence	Open-label single-arm trial	1	No
**Lyons 2018**	MDD (TRD)	15	45.4 (11.2)	26%	Severe; BDI, 34.3	Oral two doses, 10 mg and 25 mg, 7 days apart	Unavailable	Open-label single-arm trial	0	No
**Roseman 2018**	MDD (TRD)	20	44.7 (10.9)	30%	Severe; BDI, 33.7	Oral two doses, 10 mg and 25 mg, 7 days apart	Excluding psychotic disorder, serious suicide attempts, mania, and drug or alcohol dependence	Open-label single-arm trial	0	No
**Agin-Liebes 2020**	Cancer	15	53 (13.5)	60.0%	Mild; BDI, 14.1	Oral single dose, 0.3 mg/kg	Adjustment disorder and GAD	Post-RCT follow-up study	1	No
**Anderson 2020**	HIV/Cancer	18	59.2 (4.4)	0.0%	Moderate; CESD ^b^, 20.1	Oral single dose, 0.30-0.36 mg/kg	Mood disorder, anxiety disorder, and insomnia	Open-label single-arm trial	0	No
**Davis 2020**	MDD	27	39.8 (12.2)	60%	Severe, BDI, active arm, 31.9; placebo arm, 34.5	Oral two doses, 20 mg and 30 mg, 1.6 weeks apart	Excluding psychotic disorder, bipolar disorder, and drug or alcohol dependence	RCT, blinded rater	3	No

Abbreviation: AE, adverse event; BDI, Beck’s depression inventory; Dx, diagnosis; CESD, Center for Epidemiological Studies Depression Scale-Revised; GAD, generalized anxiety disorder; MDD, major depressive disorder; RCT, randomized controlled trial; TRD, treatment-resistant depression. ^a^ Definition of depression severity for BDI: minimal, 0–13; mild, 14–19; moderate, 20–28; severe, 29–63. ^b^ Definition of depression severity for CESD: 0–9; mild, 10–15; moderate, 16–24; severe, ≥25.

**Table 2:** Potential moderators for the effectiveness of psilocybin in treating depressive symptoms ^a^.

Variable	Estimate	SE	Z-Value	*p*-Value
**Dose**	−1.89	0.84	−2.25	**0.02** *
Size	−0.002	0.01	−0.17	0.86
Age	0.01	0.02	0.4	0.64
Female	0.28	0.52	0.54	0.59
Study Duration (Month)	0.02	0.07	0.33	0.74
MDD vs Cancer	−0.28	0.28	−1.02	0.31
**Two Doses vs Single Dose**	−0.50	0.26	−1.96	**0.049** *
Severity (Severe vs Non-severe)	−0.29	0.28	−1.02	0.31
RCT vs Non-RCT	0.20	0.27	0.74	0.46
With vs Without Psychotherapy	−0.37	0.33	−1.11	0.26
Placebo-controlled vs Pre-post Change	−0.27	0.30	−0.89	0.37

Abbreviation: MDD, major depressive disorder; RCT, randomized controlled trial; SE, standard error. * *p* < 0.05. ^a^ Meta-regression analyses were performed in the model of multivariate meta-analysis.

## Data Availability

The data that support the findings of the study are available from the corresponding author upon reasonable request.

## Supplementary Materials

The following supporting information can be downloaded at: https://www.mdpi.com/article/10.3390/jcm11040938/s1, Supplementary File S1. PRISMA checklist; Supplementary File S2. Searing database; Supplementary File S3. List of excluded studies after full-text screening; Figure S1. All-cause discontinuation and peak heart rate and blood pressure for psilocybin versus placebo; Figure S2. Risk of bias for included randomized controlled trials; Figure S3. Risk of bias for all included studies; Figure S4. Leave-one-out analysis for estimated Hedges’ g and heterogeneity; Figure S5. Baujat plot for included studies; Figure S6. Influence analysis for outliers of the included studies as measured by standardized residual; Figure S7. Influence analysis for outliers of the included studies as measured by Cook’s distance; Figure S8. Influence analysis for outliers of the included studies as measured by tau-squared; Figure S9. Influence analysis for outliers of the included studies as measured by hat value; Figure S10. Influence analysis for outliers of the included studies as measured by DFFITS value, which indicates (in standard deviations) how much the predicted pooled effect changes after excluding this study; Figure S11. Influence analysis for outliers of the included studies as measured by covariance ratio; Figure S12. Influence analysis for outliers of the included studies as measured by Q statistic; Figure S13. Influence analysis for outliers of the included studies as measured by weight; Table S1. Details of the results of sensitivity analyses; Table S2. Robust variance estimation for sensitivity analysis of dependent effect size estimates [42]; Table S3. Different model fitting for the trajectory of antidepressant effects of psilocybin.
