# Supplementary material for: Trajectory of Antidepressant Effects after Single- or Two-Dose Administration of Psilocybin: A Systematic Review and Multivariate Meta-Analysis
Source: J Clin Med. 2022 Feb 11;11(4):938. doi: 10.3390/jcm11040938 (PMC8879743; doi:10.3390/jcm11040938)
Supplement: Supplementary file 1 [file jcm-11-00938-s001.zip › jcm-1543729-supplementary.pdf]

Supplementary File S1. PRISMA checklist

Section and Topic	Item #	Checklist item	Location where item is reported
**TITLE**			
Title	1	Identify the report as a systematic review.	1
**ABSTRACT**			
Abstract	2	See the PRISMA 2020 for Abstracts checklist.	3-4
**INTRODUCTION**			
Rationale	3	Describe the rationale for the review in the context of existing knowledge.	5-6
Objectives	4	Provide an explicit statement of the objective(s) or question(s) the review addresses.	7
**METHODS**			
Eligibility criteria	5	Specify the inclusion and exclusion criteria for the review and how studies were grouped for the syntheses.	8-9
Information sources	6	Specify all databases, registers, websites, organisations, reference lists and other sources searched or consulted to identify studies. Specify the date when each source was last searched or consulted.	8, Appendix 1-2
Search strategy	7	Present the full search strategies for all databases, registers and websites, including any filters and limits used.	Appendix 1
Selection process	8	Specify the methods used to decide whether a study met the inclusion criteria of the review, including how many reviewers screened each record and each report retrieved, whether they worked independently, and if applicable, details of automation tools used in the process.	9, Appendix 2 and 4
Data collection process	9	Specify the methods used to collect data from reports, including how many reviewers collected data from each report, whether they worked independently, any processes for obtaining or confirming data from study investigators, and if applicable, details of automation tools used in the process.	10
Data items	10a	List and define all outcomes for which data were sought. Specify whether all results that were compatible with each outcome domain in each study were sought (e.g. for all measures, time points, analyses), and if not, the methods used to decide which results to collect.	9-10
	10b	List and define all other variables for which data were sought (e.g. participant and intervention characteristics, funding sources). Describe any assumptions made about any missing or unclear information.	9-10

Section and Topic	Item #	Checklist item	Location where item is reported
Study risk of bias assessment	11	Specify the methods used to assess risk of bias in the included studies, including details of the tool(s) used, how many reviewers assessed each study and whether they worked independently, and if applicable, details of automation tools used in the process.	10, Figures S2-S3
Effect measures	12	Specify for each outcome the effect measure(s) (e.g. risk ratio, mean difference) used in the synthesis or presentation of results.	11-12
Synthesis methods	13a	Describe the processes used to decide which studies were eligible for each synthesis (e.g. tabulating the study intervention characteristics and comparing against the planned groups for each synthesis (item #5)).	11-12
	13b	Describe any methods required to prepare the data for presentation or synthesis, such as handling of missing summary statistics, or data conversions.	10
	13c	Describe any methods used to tabulate or visually display results of individual studies and syntheses.	11-12
	13d	Describe any methods used to synthesize results and provide a rationale for the choice(s). If meta-analysis was performed, describe the model(s), method(s) to identify the presence and extent of statistical heterogeneity, and software package(s) used.	11
	13e	Describe any methods used to explore possible causes of heterogeneity among study results (e.g. subgroup analysis, meta-regression).	12
	13f	Describe any sensitivity analyses conducted to assess robustness of the synthesized results.	13
Reporting bias assessment	14	Describe any methods used to assess risk of bias due to missing results in a synthesis (arising from reporting biases).	13
Certainty assessment	15	Describe any methods used to assess certainty (or confidence) in the body of evidence for an outcome.	13-14
**RESULTS**			
Study selection	16a	Describe the results of the search and selection process, from the number of records identified in the search to the number of studies included in the review, ideally using a flow diagram.	15, Supplementary File S2
	16b	Cite studies that might appear to meet the inclusion criteria, but which were excluded, and explain why they were excluded.	Supplementary File S4

Section and Topic	Item #	Checklist item	Location where item is reported
Study characteristics	17	Cite each included study and present its characteristics.	15, Table 1
Risk of bias in studies	18	Present assessments of risk of bias for each included study.	15-16, Figure S2-S3
Results of individual studies	19	For all outcomes, present, for each study: (a) summary statistics for each group (where appropriate) and (b) an effect estimate and its precision (e.g. confidence/credible interval), ideally using structured tables or plots.	16, 18, Figure 1, Figure S1
Results of syntheses	20a	For each synthesis, briefly summarise the characteristics and risk of bias among contributing studies.	15, 16, Figure S2-S3
	20b	Present results of all statistical syntheses conducted. If meta-analysis was done, present for each the summary estimate and its precision (e.g. confidence/credible interval) and measures of statistical heterogeneity. If comparing groups, describe the direction of the effect.	16, Figure 1, Figure S1
	20c	Present results of all investigations of possible causes of heterogeneity among study results.	16-17, Table 2, Figure 2-3, Figure S4-S13
	20d	Present results of all sensitivity analyses conducted to assess the robustness of the synthesized results.	17, Figure 4, Table S1-3
Reporting biases	21	Present assessments of risk of bias due to missing results (arising from reporting biases) for each synthesis assessed.	15-16, Figure S2-S3
Certainty of evidence	22	Present assessments of certainty (or confidence) in the body of evidence for each outcome assessed.	18, Table S1-S3
**DISCUSSION**			
Discussion	23a	Provide a general interpretation of the results in the context of other evidence.	20-22
	23b	Discuss any limitations of the evidence included in the review.	23

Section and Topic	Item #	Checklist item	Location where item is reported
	23c	Discuss any limitations of the review processes used.	23
	23d	Discuss implications of the results for practice, policy, and future research.	23
**OTHER INFORMATION**			
Registration and protocol	24a	Provide registration information for the review, including register name and registration number, or state that the review was not registered.	8
	24b	Indicate where the review protocol can be accessed, or state that a protocol was not prepared.	8
	24c	Describe and explain any amendments to information provided at registration or in the protocol.	8
Support	25	Describe sources of financial or non-financial support for the review, and the role of the funders or sponsors in the review.	25
Competing interests	26	Declare any competing interests of review authors.	25
Availability of data, code and other materials	27	Report which of the following are publicly available and where they can be found: template data collection forms; data extracted from included studies; data used for all analyses; analytic code; any other materials used in the review.	26

From: Page MJ, McKenzie JE, Bossuyt PM, Boutron I, Hoffmann TC, Mulrow CD, et al. The PRISMA 2020 statement: an updated guideline for reporting systematic reviews. BMJ 2021;372:n71. doi: 10.1136/bmj.n71

For more information, visit: http://www.prisma-statement.org/

Supplementary File S2. Searching Database

Database
**MEDLINE search strategy**
1 exp Depression/ or Depression.mp. or depress*.mp. (560,150)<br>2 exp Psychological Distress/ or Psychological Distress.mp. (23,976)<br>3 exp Psilocybin/ or Psilocybin.mp. (1,105)<br>4 1 or 2 (575,825)<br>5 3 and 4 (189)
**CENTRAL search strategy**
#1 MeSH descriptor: [Psilocybin] explode all trees (75)<br>#2 (Psilocybin):ti,ab,kw (Word variations have been searched) (171)<br>#3 #1 or #2 (171)
**Embase search strategy**
#1. 'psilocybin'/exp OR psilocybin (2,002)<br>#2. 'depression'/exp OR depression (779,428)<br>#3. 'psychological distress assessment'/exp OR 'psychological distress assessment' (5,266)<br>#4. #2 OR #3 (782,461)<br>#5. #1 AND #4 (443)
**PsycINFO search strategy**
S1 depression or depressive disorder or depressive symptoms or major depressive disorder (371,918)<br>S2 psychological distress or emotional distress (37,458)<br>S3 Psilocybin (581)<br>S4 S1 OR S2 (396,283)<br>S5 S3 AND S4 (135)

Supplementary File S3. List of excluded studies after full-text screening.

First author	Title	Citation	Exclusion reason
**Euctr GB.**	Euctr GB. Assessing psilocybin as a treatment for depression.	http://wwwwhoint/trialsearch/Trial2aspx?TrialID=EUCTR2017-000219-18-GB 2018.	Study protocol
**Euctr NL.**	The Safety and Efficacy of Psilocybin in Participants with Treatment Resistant Depression.	http://wwwwhoint/trialsearch/Trial2aspx?TrialID=EUCTR2017-003288-36-NL 2018.	Study protocol
**Nct.**	Direct Comparison of Altered States of Consciousness Induced by LSD and Psilocybin.	https://clinicaltrialsgov/show/NCT03604744 2018.	Study protocol
**Nct.**	Clinical, Neurocognitive, and Emotional Effects of Psilocybin in Depressed Patients - Proof of Concept.	https://clinicaltrialsgov/show/NCT03715127 2018.	Study protocol
**Nct.**	Psilocybin vs Escitalopram for Major Depressive Disorder: comparative Mechanisms.	https://clinicaltrialsgov/show/NCT03429075 2018.	Study protocol
**Griffiths R**	Psilocybin-assisted treatment of major depressive disorder: Results from a randomized trial.	http://wwwwhoint/trialsearch/Trial2aspx?TrialID=CTRI/2019/11/022134 2019.	Conference Abstract
**Stauffer CS.**	Psilocybin-Assisted Group Therapy and Attachment: Observed Reduction in Attachment Anxiety and Influences of Attachment Insecurity on the Psilocybin Experience.	ACS Pharmacology and Translational Science 2020.	Duplicated database
**Nct.**	Psilocybin - Induced Neuroplasticity in the Treatment of Major Depressive Disorder.	https://clinicaltrialsgov/show/NCT03554174 2018.	Study protocol
**Grob C**	Psilocybin treatment for anxiety in patients with advanced-stage cancer.	Neuropsychopharmacology 2012;38:S15.	Conference Abstract
**Nct.**	The Effect of Psilocybin on MDD Symptom Severity and Synaptic Density.	https://clinicaltrialsgov/show/NCT04630964 2020.	Study protocol
**Nct**	Efficacy and Safety of Psilocybin in Treatment-Resistant Major Depression.	https://clinicaltrialsgov/show/NCT04670081 2020.	Study protocol
**Reynolds CF.**	Psilocybin-Assisted Supportive Psychotherapy in the Treatment of Major Depression - Quo Vadis?	Cutaneous and ocular toxicology 2014; 33: 54-9.	Insufficient data despite of request
**Nct.**	A Two-Year Observational Follow-up Study of Subjects With Major Depressive Disorder Following a Randomized, Double-Blind Single-Dose of Psilocybin or Niacin-Control.	https://clinicaltrialsgov/show/NCT04353921 2020.	Study protocol

First author	Title	Citation	Exclusion reason
**Roseman L**	Increased amygdala responses to emotional faces after psilocybin for treatment-resistant depression.	Neuropharmacology 2018;142:263-9.	No outcome of interest

Figure S1. All-cause discontinuation and peak heart rate and blood pressure for psilocybin versus placebo.

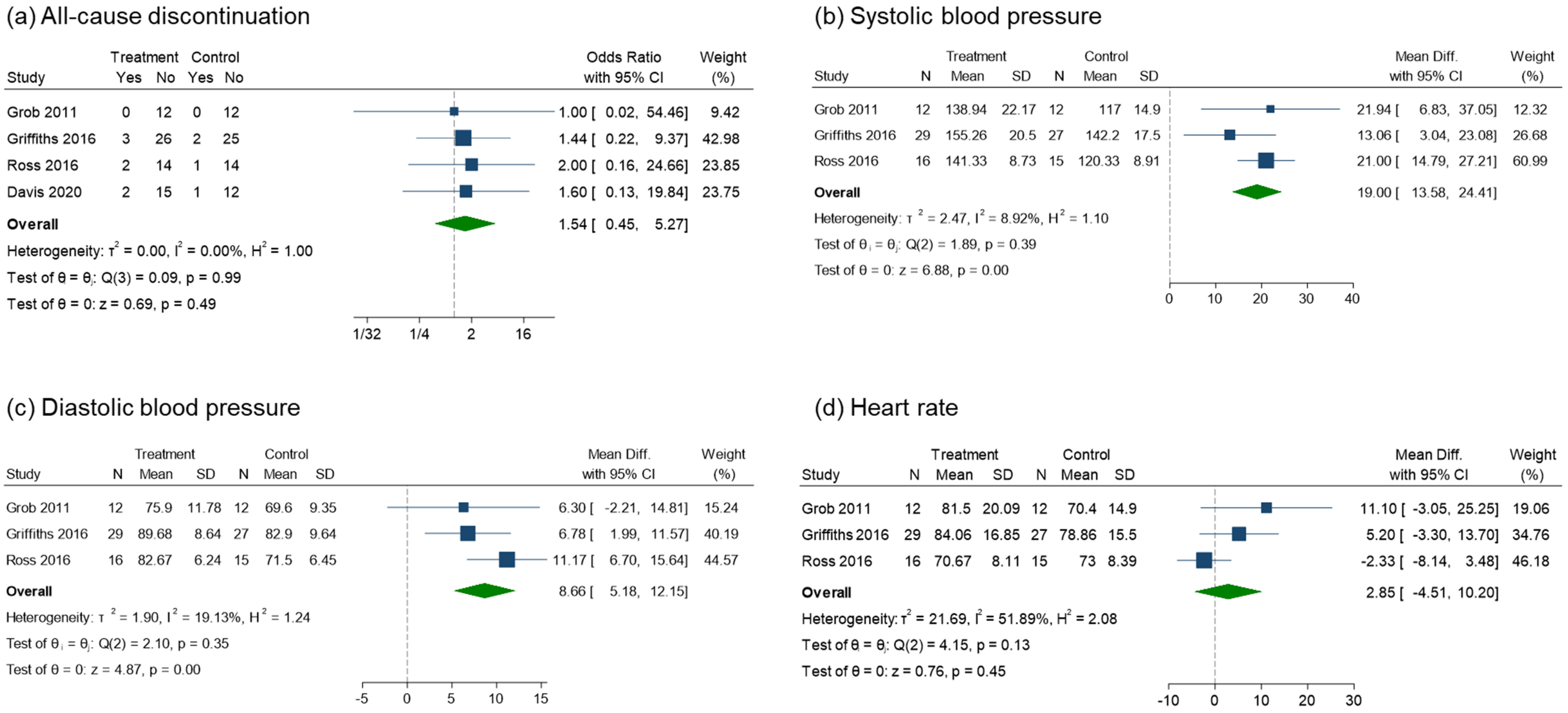

Figure S2. Risk of bias for included randomized controlled trials.

(a) Risk of Bias Summary

(b) Risk of Bias Graph

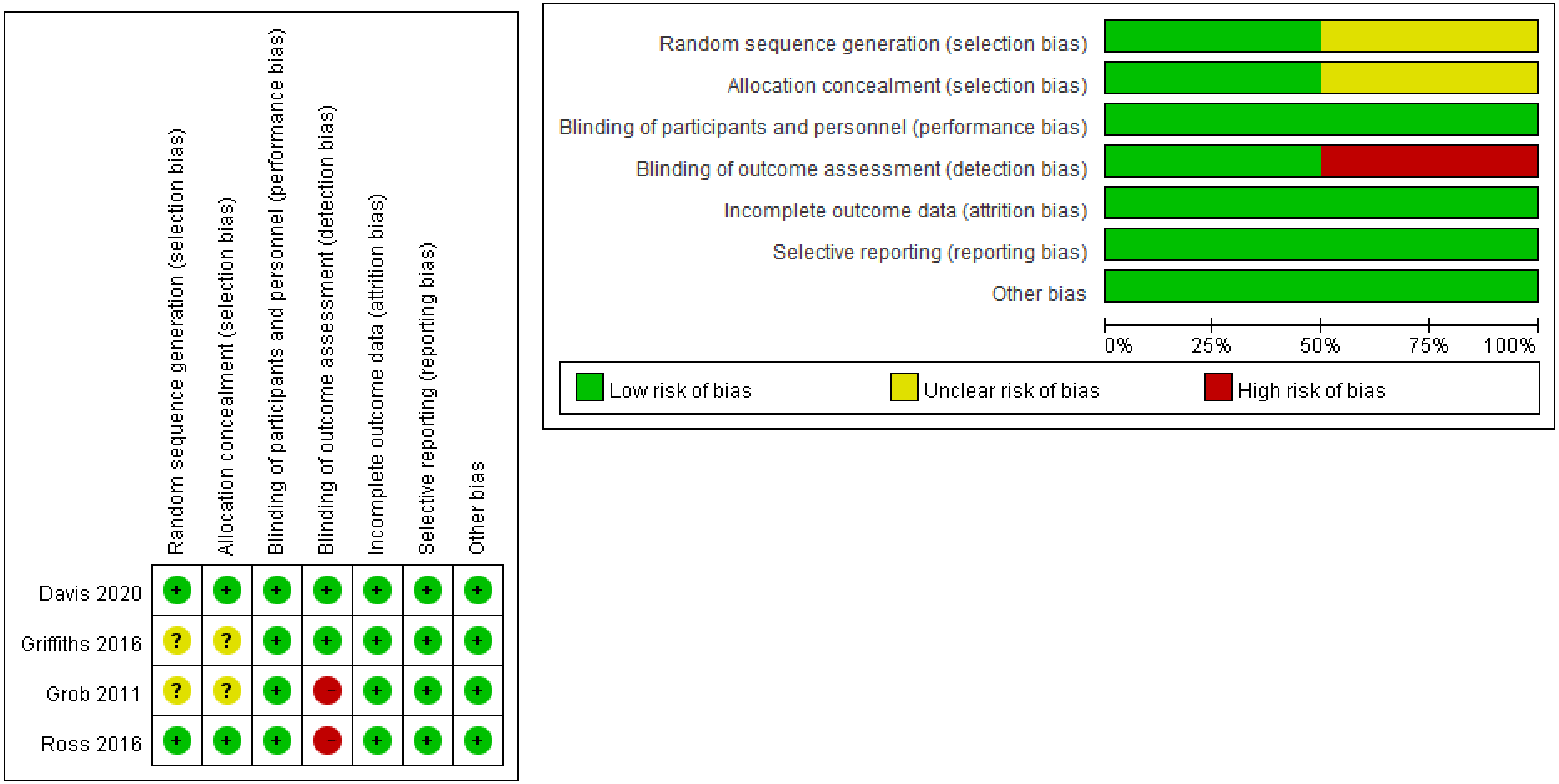

Figure S3. Risk of bias for all included studies.

(b) Risk of Bias Summary

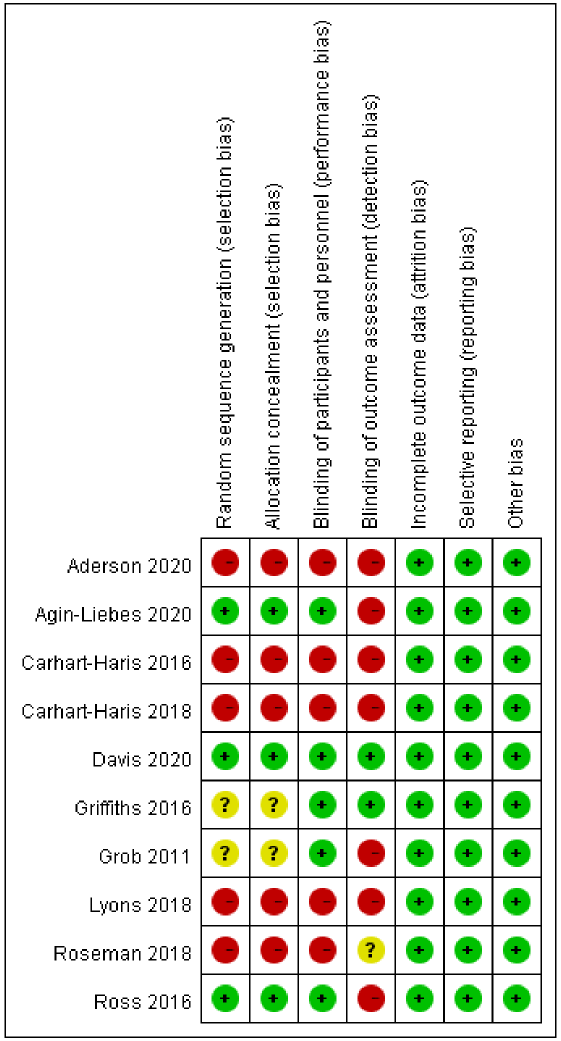

(b) Risk of Bias Graph

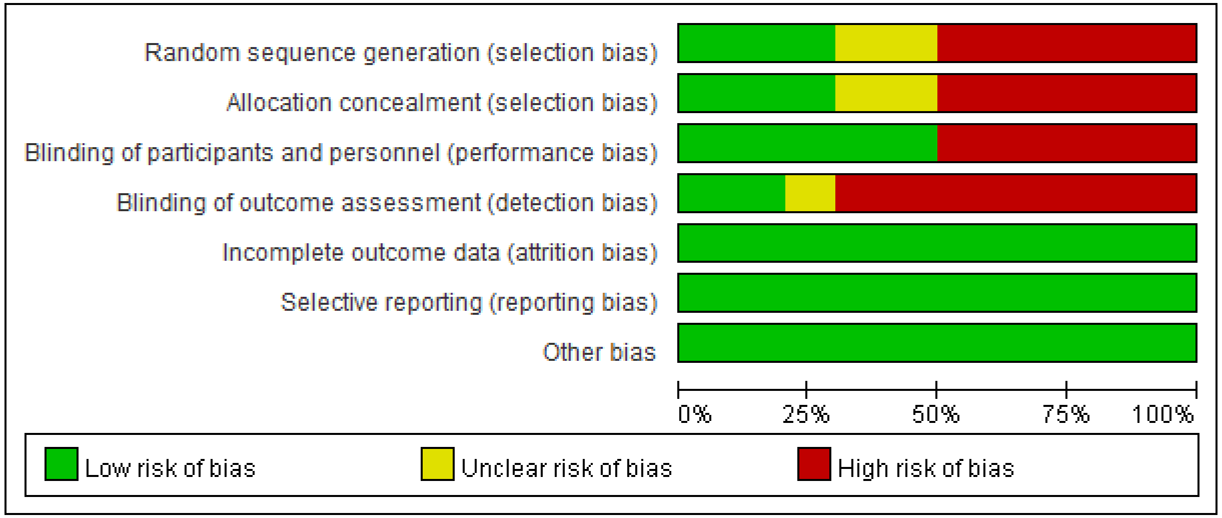

Figure S4. Leave-one-out analysis for estimated Hedges' g and heterogeneity.

(A) Hedges ' g

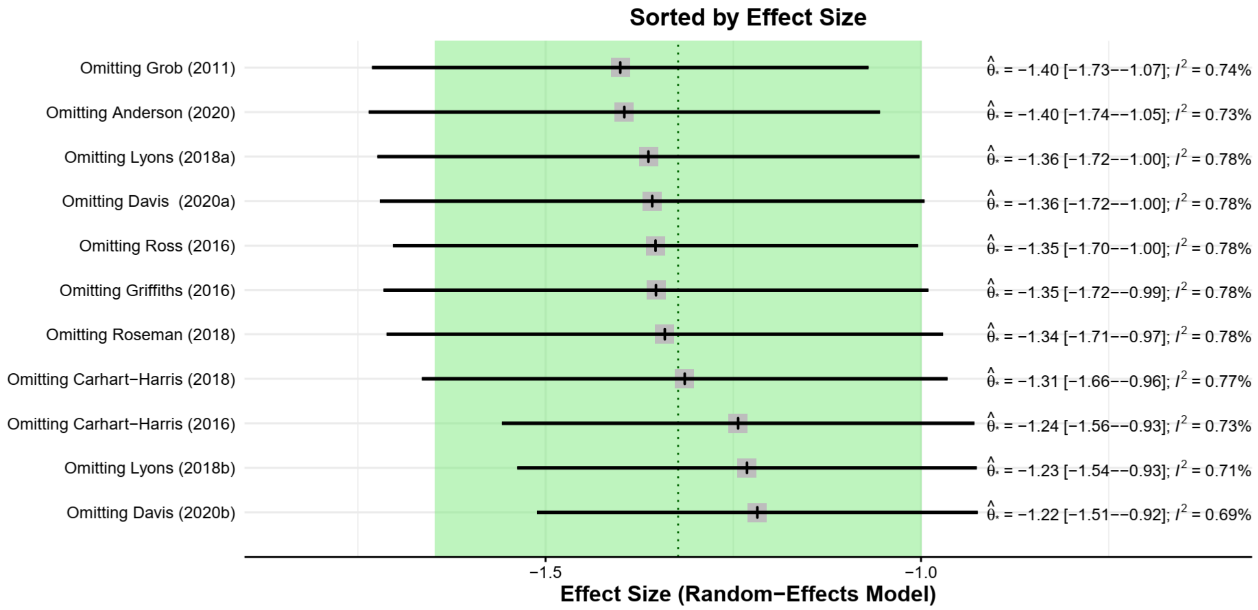

(B) Heterogeneity (I-squared)

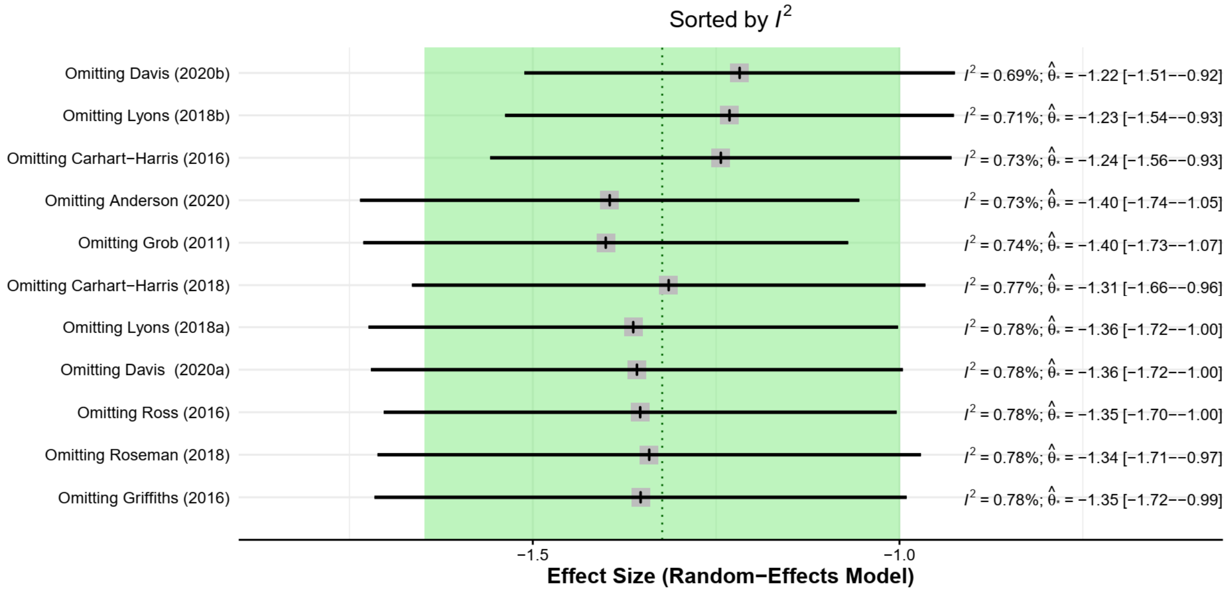

Figure S5. Baujat plot for included studies.

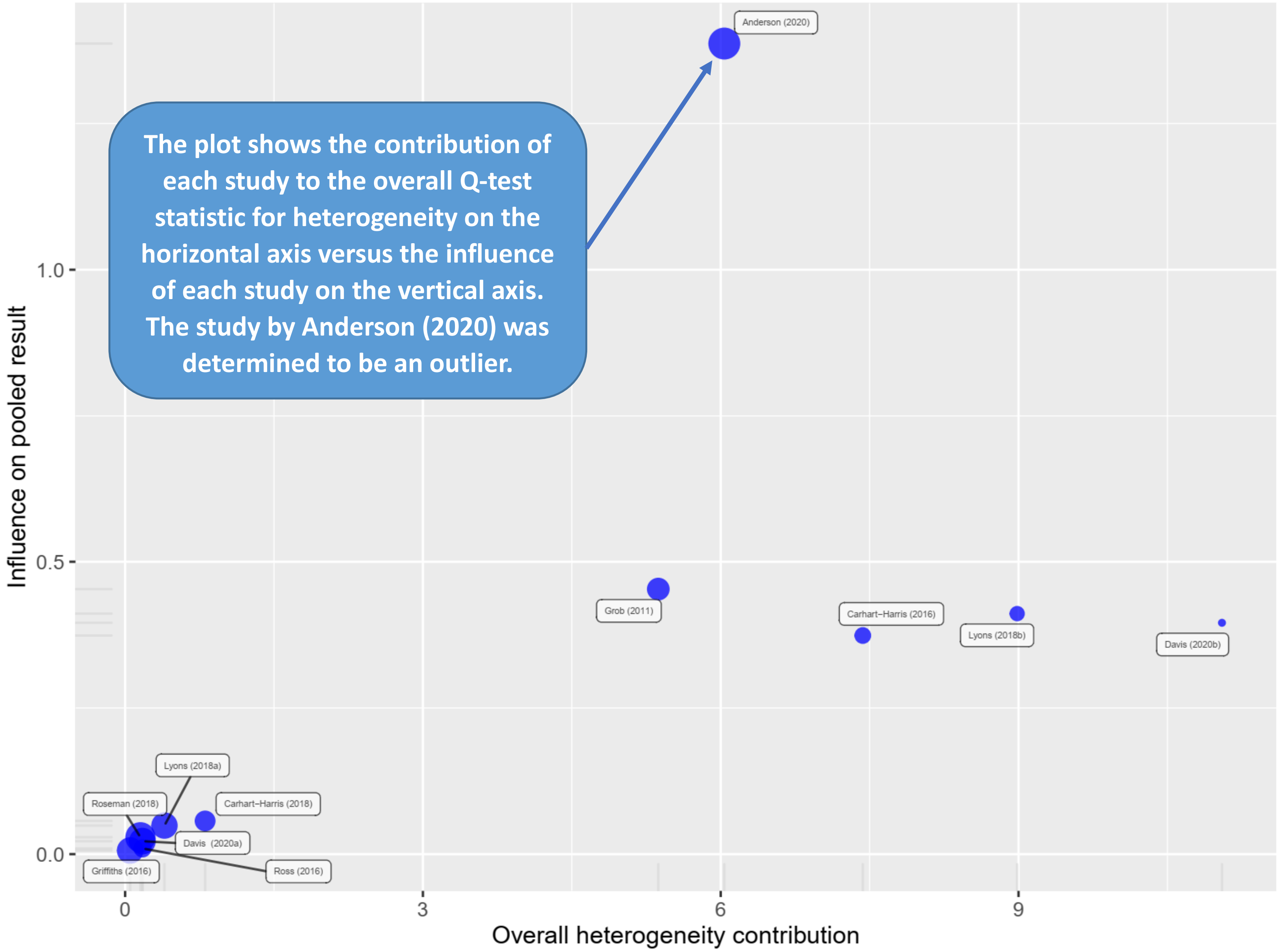

Figure S6. Influence analysis for outlier of the included studies as measured by standardized residual.

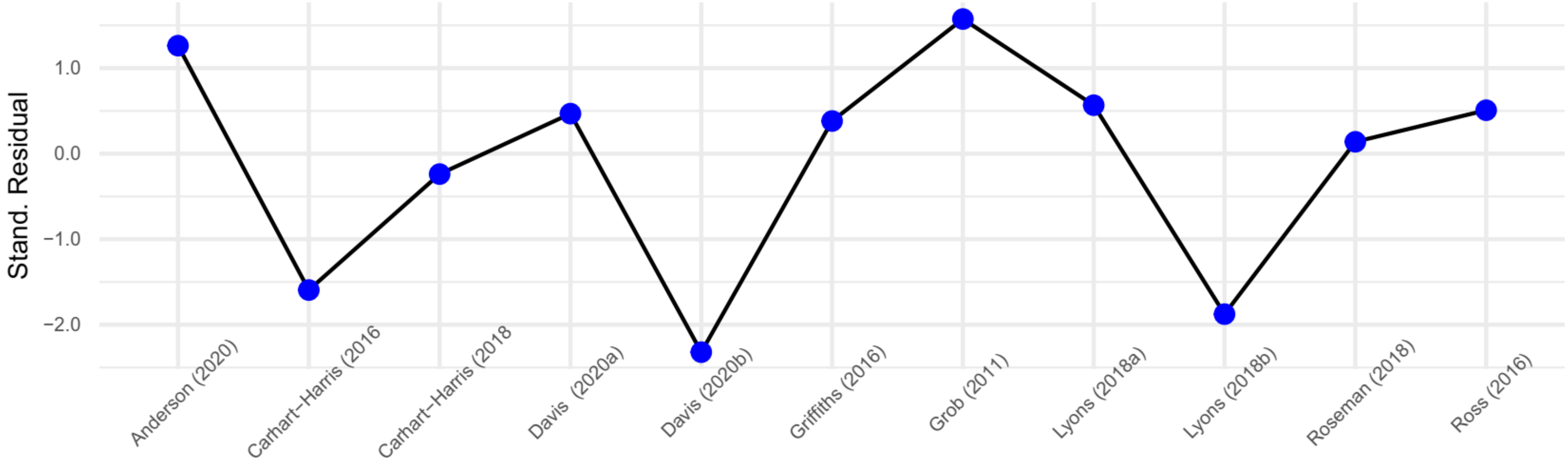

If a study was determined being an influential study, its value will be displayed in red.

Figure S7. Influence analysis for outlier of the included studies as measured by Cook's distance.

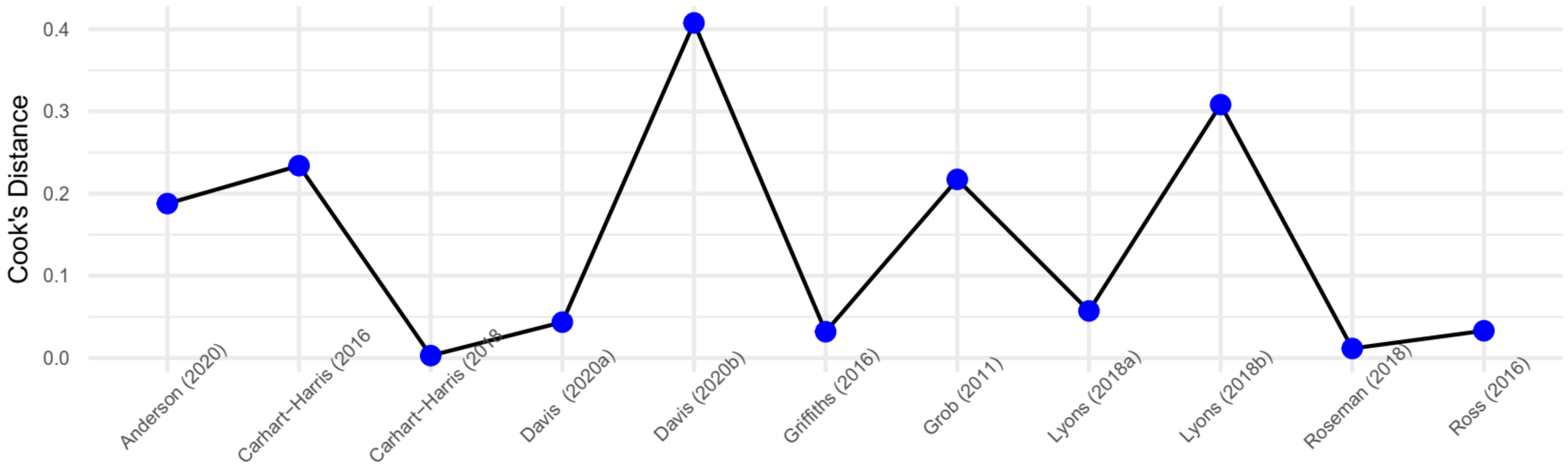

If a study was determined being an influential study, its value will be displayed in red.

Figure S8. Influence analysis for outlier of the included studies as measured by tau-squared.

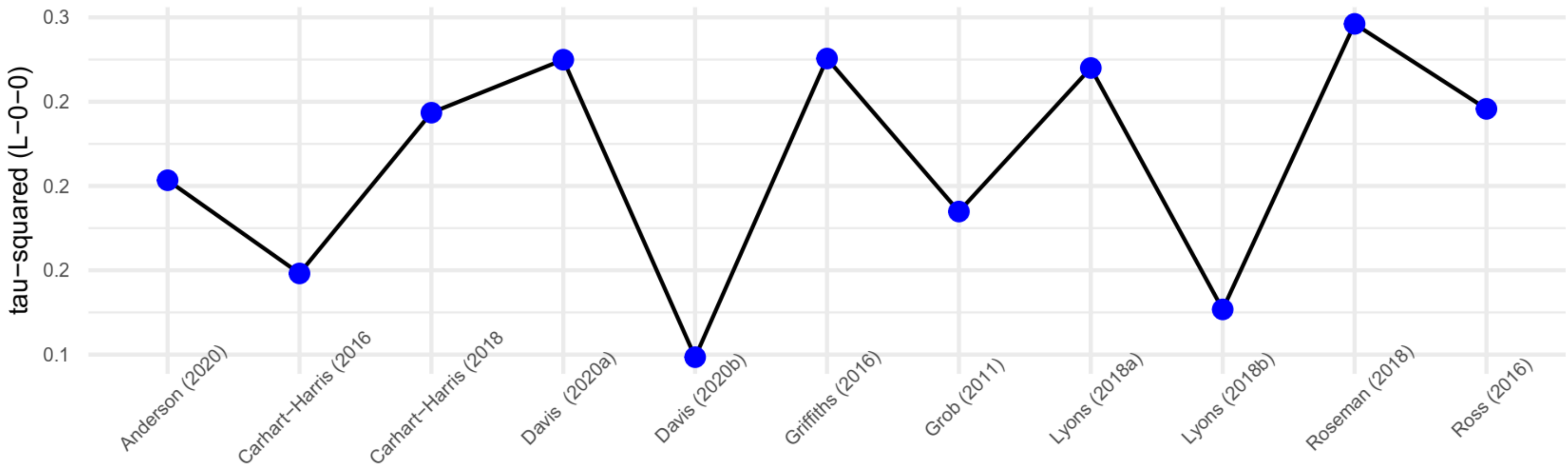

If a study was determined being an influential study, its value will be displayed in red.

Figure S9. Influence analysis for outlier of the included studies as measured by hat value.

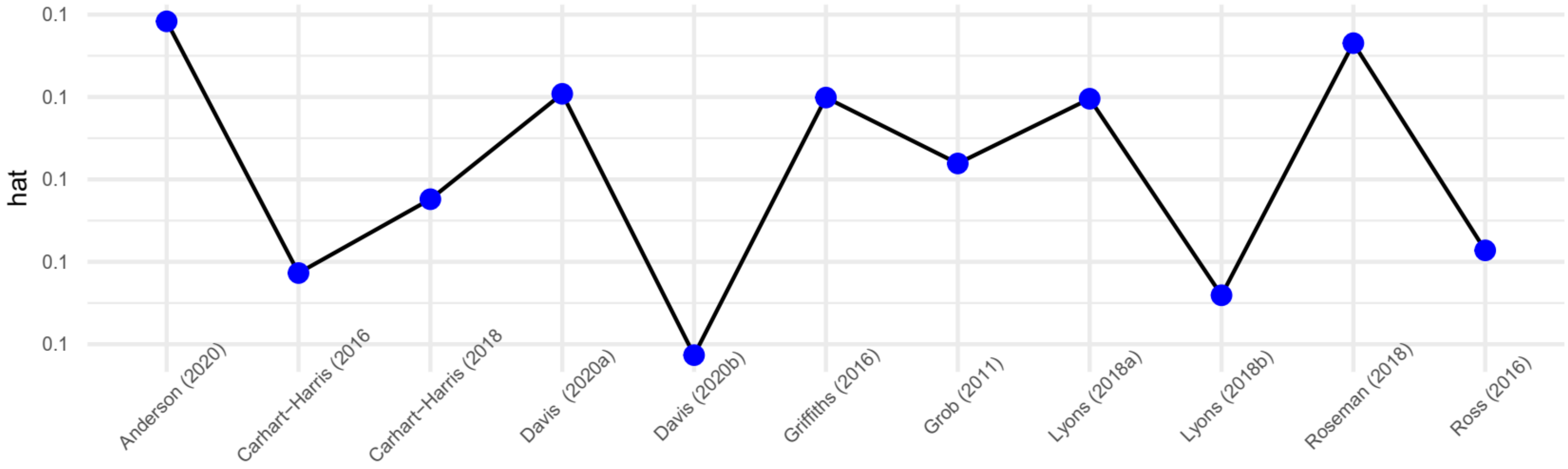

If a study was determined being an influential study, its value will be displayed in red.

Figure S10. Influence analysis for outlier of the included studies as measured by DFFITS value, which indicates in standard deviations how much the predicted pooled effect changes after excluding this study.

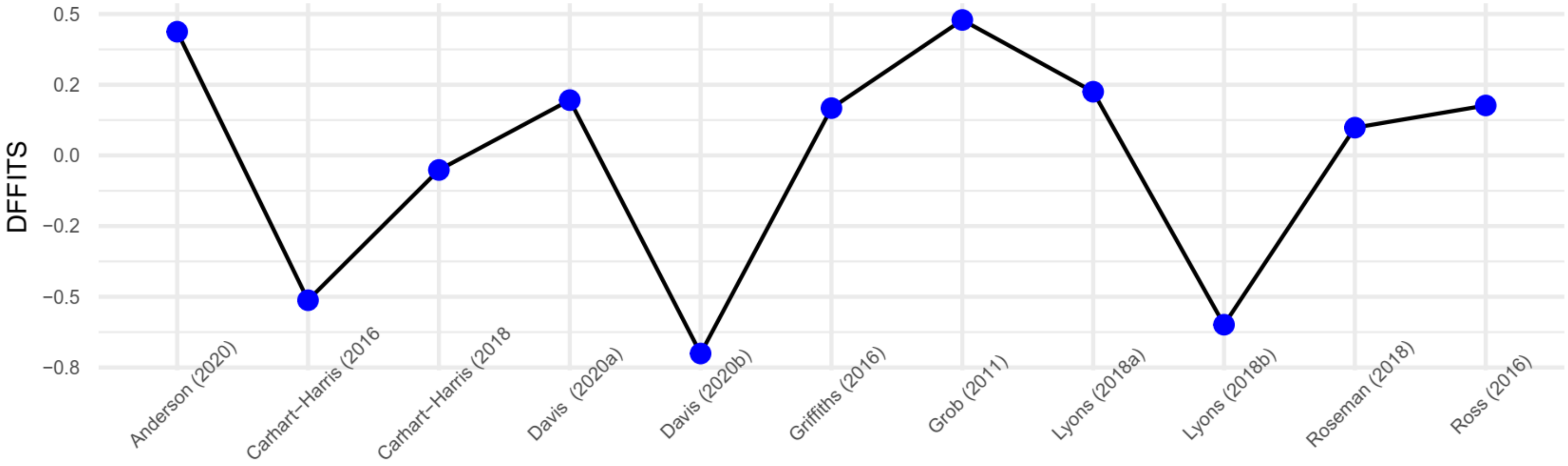

If a study was determined being an influential study, its value will be displayed in red.

Figure S11. Influence analysis for outlier of the included studies as measured by covariance ratio.

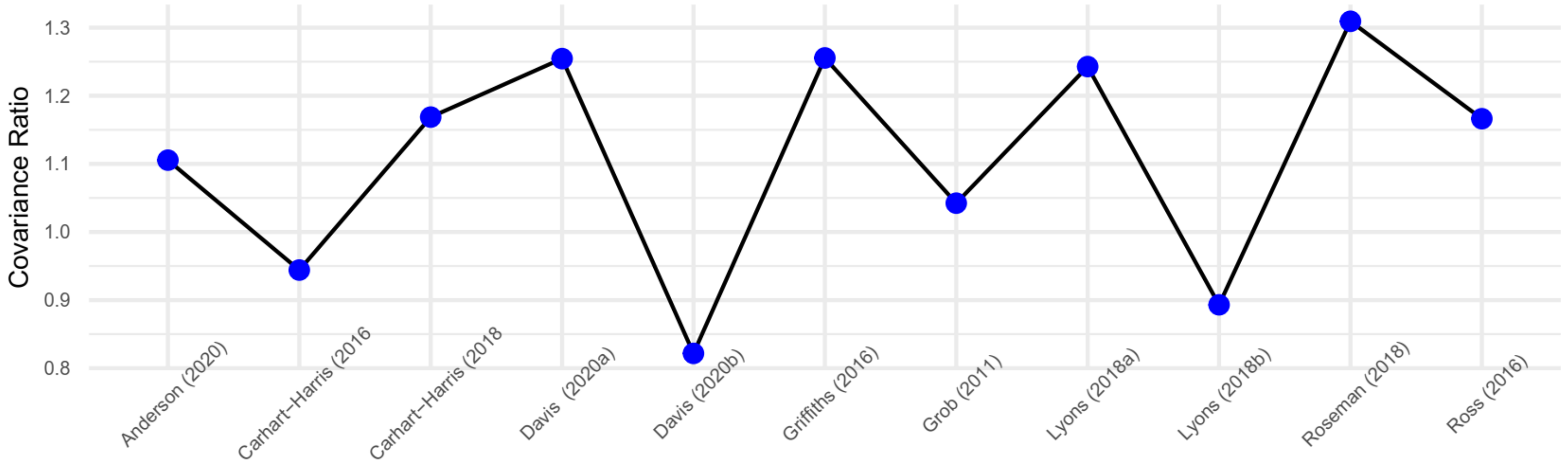

If a study was determined being an influential study, its value will be displayed in red.

Figure S12. Influence analysis for outlier of the included studies as measured by Q statistic.

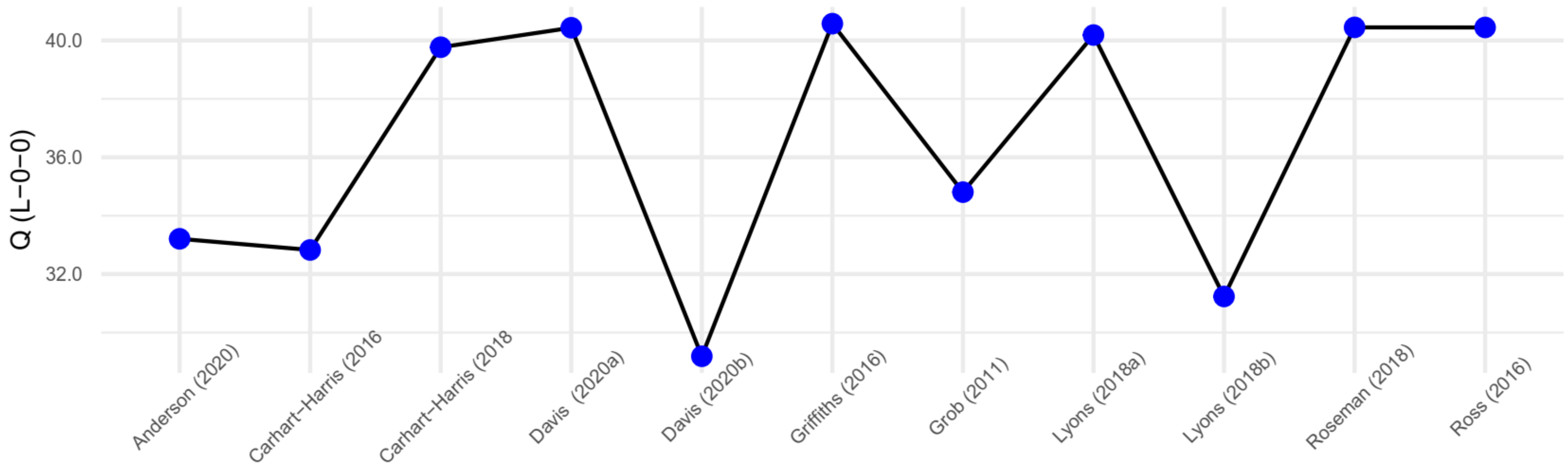

If a study was determined being an influential study, its value will be displayed in red.

Figure S13. Influence analysis for outlier of the included studies as measured by weight.

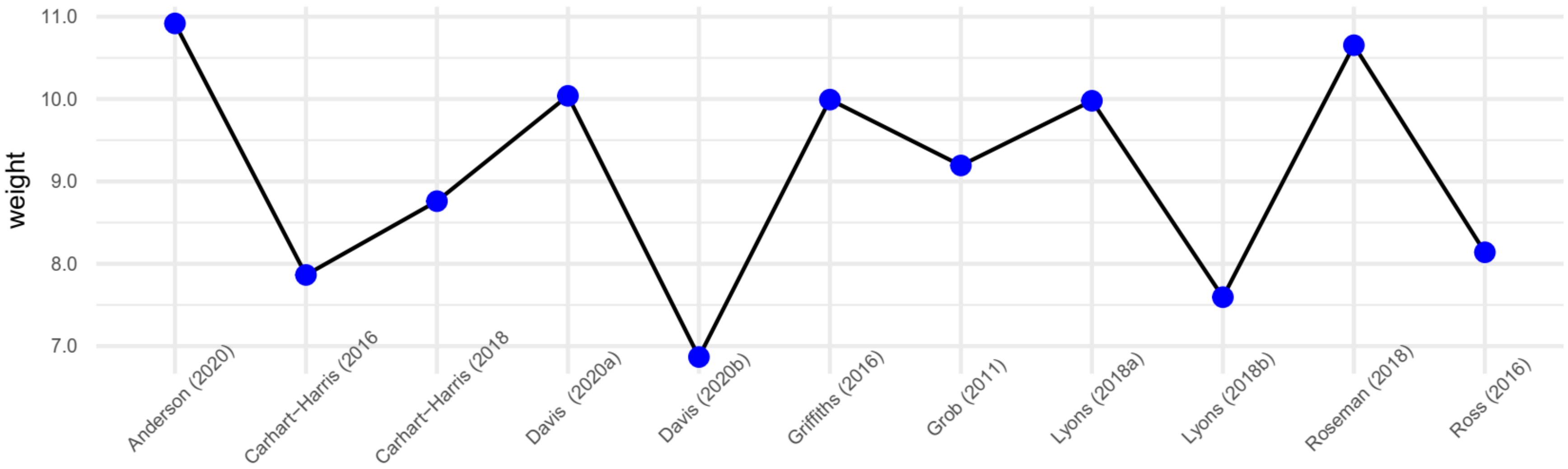

If a study was determined being an influential study, its value will be displayed in red.

Table S1. Details of the results of sensitivity analyses.

Analysis	Day 1	Week 1	Month 1	Month 3	Month 6
Raw Model	-0.75; -1.15, -0.35	-1.74; -2.15, -1.32	-1.35; -1.78, -0.93	-0.91; -1.31, -0.51	-1.12; -1.56, -0.68
RCT	-0.50; -1.18, 0.17	-1.90; -2.84, -0.95	-1.40; -2.20, -0.61	-0.95; -1.83, -0.06	-1.23; -2.03, -0.44
Non-RCT	-1.21; -1.74, -0.68	-1.67; -2.08, -1.27	-1.37; -1.84, -0.89	-0.95; -1.34, -0.56	-0.94; -1.45, -0.44
Single Dose	-0.56; -0.92, -0.27	-1.39; -1.89, -0.90	-0.70; -1.13, -0.26	-0.63; -0.99, -0.28	-1.09; -1.48, -0.71
Two Doses	-1.00; -1.86, -0.15	-2.20; -2.84, -1.57	-1.94; -2.63, -1.26	-1.22; -1.95, -0.49	-1.08; -1.94, -0.23
MDD	-0.78; -1.46, -0.11	-1.92; -2.43, -1.41	-1.81; -2.47, -1.17	-1.15; -1.86, -0.45	-1.04; -1.87, -0.21
Cancer/HIV	-0.76; -1.13, -0.38	NA	-0.76; -1.15, -0.38	-0.67; -1.00, -0.34	-1.17; -1.52, -0.81
Excluding Anderson 2020	-0.77; -1.24, -0.30	-1.76; -2.20, -1.32	-1.39; -1.84, -0.93	-0.96; -1.43, -0.49	-1.14; -1.62, -0.66
Studies ≥ 4 F/Us	-1.21; -1.91, -0.50	-1.17; -1.84, -0.49	-1.11; -1.75, -0.47	-0.65; -1.26, -0.04	-0.89; -1.50, -0.27

[a] represented by Hedges' g with 95% confidence interval.

[b] Raw model: the original model; RCT: using data on RCTs (definitely no duplicate datasets); Non-RCT: using data on non-RCTs; Single dose: using studies with single dose of administration; Two doses: using studies with two doses of administration; MDD: using studies with MDD participants; Cancer/HIV: using studies recruiting patients with cancer or HIV infection; Excluding Anderson 2020: a statistical model excluding the study by Anderson et al, 2020; Studies ≥ 4 F/Us: using studies with at least four time-point follow-up measurements.

Table S2. Robust variance estimation for sensitivity analysis of dependent effect size estimates.

Variable	Coefficient estimate	Standard error	t-statistic	Df	P-value
factor(time)1	-0.751	0.238	-3.16	5.19	0.02377*
factor(time)2	-1.735	0.234	-7.4	6.46	< 0.001***
factor(time)3	-1.352	0.265	-5.11	5.69	0.00257**
factor(time)4	-0.912	0.151	-6.05	5.39	0.00136***
factor(time)5	-1.117	0.113	-9.92	4.29	< 0.001***

[a] We used vcov = "CR2" option for the variance-covariance matrix, meaning that the standard errors will be corrected using the bias-reduced linearization estimator proposed by Bell and McCaffrey (2002) and further developed in Pustejovsky and Tipton (2018).

Reference

1. Bell, R. M., & McCaffrey, D. F. (2002). Bias reduction in standard errors for linear regression with multi-stage samples. Survey Methodology, 28(2), 169-181.
2. Pustejovsky, J. E. & Tipton, E. (2018). Small sample methods for cluster-robust variance estimation and hypothesis testing in fixed effects models. Journal of Business and Economic Statistics, 36(4), 672-683.

Table S3. Different model fitting for the trajectory of antidepressant effects of psilocybin.

Variable	Original model	Linear model (month)	Quadratic model	Restricted cubic splines model
Plot	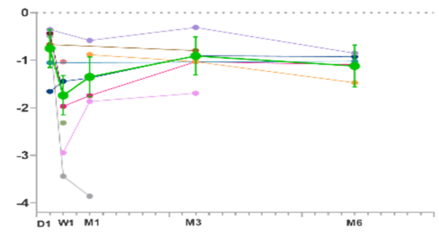	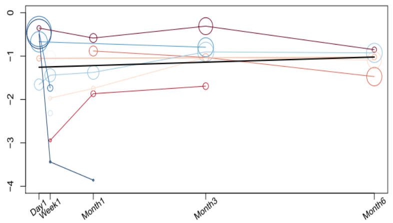	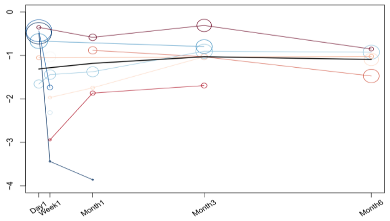	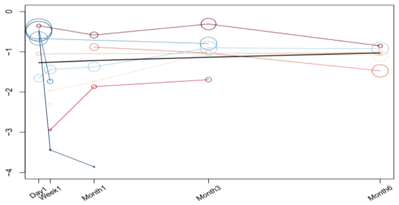
LogLik	-21.75	-29.82	-29.26	-29.45
Deviance	43.49	59.64	58.53	58.90
AIC	57.49	67.64	68.53	68.90
BIC	66.02	72.97	75.01	75.37
AICC	64.08	69.38	71.38	71.75
P-value	Reference	0.0011**	0.0005***	0.0005***
P-value	-	Reference	0.387	0.291
P-value	-	-	Reference	<0.001***

[a] P-value indicates a comparison of model fitting with the reference model.

Abbreviation: AIC, Akaike information criterion; AICC, Akaike information criterion corrected for small sample sizes; BIC, Bayesian information criterion; LogLik, log-likelihood.
